# Digital Archiving of Direct-to-Consumer Marketing Claims Made by Businesses Selling Unproven Stem Cell Interventions

**DOI:** 10.2196/95156

**Published:** 2026-10-23

**Authors:** Leigh Turner

**Affiliations:** 1 Department of Health, Society, and Behavior, School of Population and Public Health, University of California, Irvine, 856 Health Sciences Road, Irvine, California, CA, 92617, United States, 1 949-824-5735

**Keywords:** internet-based direct-to-consumer marketing, stem cells, digital archiving, unproven, ethics

## Abstract

Over the last 2 decades, a large global marketplace has emerged in which businesses use direct-to-consumer advertising to sell putative stem cell treatments that are unsupported by convincing scientific evidence of safety and efficacy. It is common for such purported stem cell therapies not to have been reviewed and approved by national regulatory authorities, even though they are supposed to undergo rigorous premarketing review by regulatory bodies responsible for evaluating such medical products and determining whether they are backed by sufficient evidence to warrant approval. The marketing claims, commercial activities, and clinical practices of such businesses have resulted in patients experiencing serious harms, including loss of life, physical injuries, financial losses, and avoidable delays in accessing evidence-based treatments. Such businesses are also responsible for collective harms, including diverting patients from participating in legitimate stem cell clinical studies, damaging the field of credible stem cell research, and contributing to public confusion and misunderstandings about how to distinguish evidence-based stem cell therapies from stem cell interventions that are not backed by robust data. Researchers in bioethics, health law, the social sciences, and other academic fields have made important contributions to the critical analysis of direct-to-consumer digital marketing claims made by businesses selling unproven and unapproved stem cell interventions. Scholars studying such marketing representations have documented the use of persuasive “tokens of legitimacy” to make advertised stem cell interventions seem credible, trustworthy, and backed by current scientific evidence. Acknowledging the value of such scholarship, there is a growing need for the development of best practices in research methods and data-archiving practices. In particular, there is a need for increased digital archiving of online marketing claims made by businesses selling supposed stem cell treatments. Numerous parties can benefit from increased digital archiving of such problematic marketing representations. However, analyzing, archiving, and critiquing the marketing claims, business operations, and clinical practices of such businesses must be done in a careful, rigorous, and evidence-based manner that is attentive to the intimidating tactics some of these businesses use when responding to public criticism. Researchers need to document the marketing representations made by businesses selling unapproved and unproven stem cell interventions in ways that minimize exposure to legal threats and other efforts to suppress critical analysis. Using the Wayback Machine or other comparable archiving tools to create digital repositories of such marketing representations is one way to mitigate such threats, by capturing the exact claims such businesses make when promoting putative stem cell therapies. Archiving such digital marketing representations is important for academic research and can play a valuable role in other contexts, such as in legal actions taken against businesses using purportedly deceptive marketing claims to sell unproven and unapproved stem cell-based interventions.

## Introduction

For approximately 2 decades, bioethicists, social scientists, health researchers, and other scholars have analyzed and critiqued direct-to-consumer marketing claims made by businesses selling purported stem cell treatments for pain, orthopedic diseases and injuries, neurological conditions, respiratory diseases, sports injuries, immunological conditions, cardiovascular diseases, vision loss, and other indications [1-5]. With its potentially global reach, the internet is a crucial marketing platform for businesses selling putative stem cell therapies, though companies also promote stem cell products using other modes of communication such as billboards, printed flyers, in-person sales pitches, and newspaper, radio, and television advertising [6-15]. Digital marketing has the advantage of enabling such businesses to target local clients while also attracting customers from more distant regions. Recognizing the reach and importance of online advertising, researchers have studied the digital marketing platforms of such businesses, analyzing company websites, social media feeds such as Facebook and Twitter (subsequently rebranded X), YouTube videos, and other forms of advertising [4,6,10,12,16-21].

Studies analyzing the online marketing representations of such businesses range from efforts to investigate the entire global direct-to-consumer marketplace within which particular businesses operate to focused surveys of specific national or regional markets for selling purported stem cell therapies [4,10,11,22]. For example, researchers have studied direct-to-consumer marketing of stem cell interventions by businesses located in such countries as Australia, Canada, Japan, Panama, Poland, and the United States, as well as along the US-Mexico border [6,12,21,23-35]. These studies provide insight into the operations of a global marketplace for such products as well as the distinctive features of regional markets. Temporal analyses reveal how such markets are dynamic, expanding or shrinking over time, and responsive to various factors, including changes in applicable regulatory frameworks, actions by regulators, and the lack of activity by bodies responsible for enforcing laws and regulations [30,36].

Despite the substantial body of social science studies, ethical analyses, and legal critiques of marketing claims made by businesses selling purported stem cell therapies, researchers have to date given only limited consideration to the question of what constitutes best practices in data mining of business websites, analyses of marketing claims, and archiving of digital marketing representations. Absent the development of widely recognized standards and a broad consensus concerning what constitutes best practices when conducting such research and capturing primary source material, researchers have used various approaches to analyze, document, and archive such marketing claims. Appreciating the value of a plurality of research tools, theoretical frameworks, and methodologies, future research in this area would benefit from greater self-reflection and scholarly discussion concerning best practices in scholarly methods, and at least some degree of standardization concerning optimal research methods, analytic tools, and data archiving practices.

Preserving archival records of direct-to-consumer marketing claims made by businesses selling supposed stem cell therapies is important given the fluid nature of this marketplace, the regular changes many businesses make to their digital marketing claims, and the frequent disappearance from the internet of individual web pages and entire websites [36]. It can be difficult to track, document, and understand such temporal changes in advertising representations if digital marketing claims are not captured and archived by researchers. Furthermore, researchers are vulnerable to challenges concerning the accuracy of their findings if businesses change their marketing claims after scholars have completed their analyses of online marketing claims, and researchers have not archived the digital marketing representations that constitute the evidence base for their scholarship. Digital archiving of such marketing claims can help researchers defend their work from criticism, capture representations that often change over time, and better document what kinds of problematic marketing representations such businesses make. Publicly archiving such digital representations can benefit researchers, journalists, regulators, litigants, and other parties, though at present there is considerable variation in whether scholars make such material publicly accessible, maintain digital records that are not publicly shared, or do not archive primary source material.

## Harms Resulting From Direct-to-Consumer Marketing of Purported Stem Cell Therapies

One compelling reason for analyzing and archiving the digital marketing representations of businesses engaged in direct-to-consumer advertising of purported stem cell therapies is that such practices have resulted in significant harm. Researchers examining the marketing representations, commercial activities, and clinical practices of businesses marketing purported stem cell therapies have documented numerous harms resulting from businesses using digital marketing platforms and other types of advertising to sell medical products that have not been approved by national regulators and are unsupported by convincing scientific evidence of safety and efficacy for specific clinical uses [24,37-39].

Numerous patients have experienced serious adverse events or significant complications after being administered unapproved and unproven stem cell products that were marketed as treatments but instead harmed recipients [24,37-44]. There are also patients who have experienced consequential financial losses after being misled by deceptive marketing representations [33,45,46]. Some patients who have alleged that they were deceived by the advertising claims of such businesses and, as a result, experienced injuries or financial losses have, in civil litigation, also claimed that they experienced emotional harms, psychological harms, or other kinds of harm. For example, pursuing unproven and unapproved medical interventions can result in delays in patients accessing evidence-based medical interventions.

In addition to cases where individual patients have been harmed, such activity has resulted in social harms. Such collective harms can be difficult to quantify, but they are worth noting even if they are not easily measured and monitored. For example, many stem cell researchers and other scholars are concerned that the field of legitimate stem cell research is vulnerable to having its reputation damaged by the activities of businesses that use misleading marketing representations to sell unproven and unapproved stem cell interventions [15,47,48]. Such researchers have expressed concerns that physical injuries to patients or significant financial losses experienced by patients exposed to deceptive advertising claims could damage public trust in credible stem cell research and harm the field. Some patients who might have otherwise decided to participate in well-designed and carefully conducted clinical trials testing investigational stem cell-based interventions have likely instead been diverted by businesses using appealing marketing messages to frame unproven interventions as safe and effective “stem cell treatments.” Diversion of patients from opportunities to participate in rigorous clinical trials can undermine efforts to recruit research participants and make it more difficult to conduct such studies. Other businesses promote “pay-to-participate stem cell studies” that have significant scientific and ethical shortcomings and are unlikely to generate high-quality safety and efficacy data [49]. The promotion of such studies can also have the effect of harming public understanding of stem cell research, contributing to stem cell-related misinformation, and making it difficult for members of the public to distinguish well-designed clinical trials from studies that are little more than marketing devices.

Acknowledging the many sources of health-related misinformation to which patients and other members of the public are exposed, public understanding of stem cells has likely worsened as a result of the many businesses circulating misleading marketing claims about stem cells and stem cell therapies [6,50-55]. The use of digital marketing to promote inaccurate stem cell-related claims has probably contributed to public confusion about how to distinguish evidence-based stem cell therapies from interventions unsupported by robust safety and efficacy data. Scientific societies such as the International Society for Stem Cell Research (ISSCR) have responded to such deceptive marketing claims about stem cells by helping patients spot “red flags” in advertising, explaining the importance of testing investigational stem cell products in clinical trials, urging scientists to avoid using stem cell hyperbole that helps feed misleading marketing claims about stem cells, and developing thoughtful guidelines for basic science research and translational clinical research [15,56-59]. Despite these efforts, the widespread use of marketing misrepresentations must make it difficult for members of the public to make distinctions among evidence-based and approved stem cell-based interventions, investigational stem cell products currently being tested in legitimate clinical trials, and unapproved and unproven stem cell-based products promoted as therapies [6,50,53,54].

## Studying Direct-to-Consumer Marketing of Purported Stem Cell Therapies

Researchers have taken different approaches when analyzing digital marketing representations on company websites and other platforms and archiving such promotional claims [3-5,7,10-12,18,20,22,26-30,32,33,47,60-63]. For example, some researchers have, in their publications and public outreach activities, identified by name specific businesses engaged in such commercial activity. However, identifying particular businesses and criticizing them for using misleading marketing claims poses risks and can expose researchers and other commentators to intimidating responses from such businesses [64,65]. Such responses can include calls for corrections, retractions, or even threats of legal action [66]. This tactic of attempting to intimidate critics is part of a broader phenomenon in which lawsuits or threats concerning the possibility of litigation are used to suppress critical analysis.

Stem cell societies have also been faced with such threats. For example, ISSCR was confronted with this challenge years ago when planning to flag businesses selling purported stem cell treatments without appropriate oversight and proper regulatory approval [15,67,68]. ISSCR created a “Submit a Clinic” website and encouraged patients and other individuals to provide the names of businesses selling supposed stem cell therapies. Using these leads, ISSCR had planned to contact identified businesses and ask them to answer questions about whether they were subject to review by independent oversight bodies such as ethics review committees or institutional review boards and were regulated by authorized regulatory bodies [15,67]. ISSCR planned to share the responses it received on its website. However, this effort to gather information and promote transparency concerning the extent to which such businesses were subject to meaningful regulatory oversight reportedly resulted in ISSCR receiving responses from lawyers representing such businesses. Concerned about the threat of litigation, ISSCR decided not to proceed with this effort [67]. While this decision is understandable given the cost of having to defend against such lawsuits, it also reveals how challenging it can be to publicly criticize businesses using marketing misrepresentations to sell purported stem cell treatments.

Scholars writing about such businesses have also encountered threatening responses from companies that do not want to have their marketing claims and business practices exposed and publicized. Engaging in such scholarship comes with risks, in environments where academic journals and research institutions typically want to mitigate such risks and avoid being subject to litigation. Perhaps in part to reduce the risks associated with describing particular businesses by name while critiquing their marketing representations and clinical activities, some researchers have not specified in their publications the identities of such businesses. Rather, they have addressed in more general terms ethical, legal, and scientific concerns related to deceptive direct-to-consumer marketing of purported stem cell therapies.

Other differences exist in the approaches researchers have taken when analyzing businesses engaged in direct-to-consumer marketing of putative stem cell therapies. Some researchers have built extensive databases capturing a variety of digital marketing representations made by such businesses. In contrast, other researchers appear to have identified and monitored many such businesses and provided insightful critiques of their promotional claims without constructing large databases using social science research methods that analyze a broad range of marketing representations. Some of these differences in approaches might be attributable, at least in part, to the particular fields of study within which researchers situate themselves, the specific research methods in which such scholars have been trained, and the amount of funding researchers have to support such scholarship.

There is likewise considerable variation in archiving practices. Some researchers have created archival records of the businesses they have studied by taking screenshots of digital marketing claims, preserving printed copies or electronic records of digital marketing claims, or otherwise capturing and preserving digital marketing claims. Other researchers have critiqued misleading marketing claims without addressing whether they created digital or print archives documenting such representations.

Researchers have also differed in whether they make their primary datasets open access and publicly available with their published studies, publish redacted versions without company names or other identifiers, do not provide public access to such data, or make their research databases available to other researchers only if specific conditions are met. To date, standards for archiving such primary source material and for determining what information should be made publicly available have not yet emerged. Research teams have taken different approaches when archiving digital representations and determining whether such information should be made publicly accessible.

It might seem obvious to proponents of open science that researchers should have to make complete, unredacted copies of their databases publicly available. However, this emphasis on transparency and disclosure does not grapple sufficiently with the extent to which such businesses can pose significant legal and financial threats to researchers and the journals in which they publish their work. Though researchers should be able to defend themselves against accusations of libel if they have described businesses’ marketing claims in an accurate, truthful, and evidence-based manner, the financial costs associated with responding to such allegations can be substantial. It was the threat of incurring such significant legal expenses that reportedly informed ISSCR’s decision not to proceed with its “Submit a Clinic” initiative.

Another concerning risk associated with making such databases fully open access is that if researchers have identified businesses by name, provided their website addresses, and otherwise gathered detailed information about such businesses’ commercial practices and marketing claims, this data can then be acquired and repurposed by companies that aggregate such information and use it to connect patients to such businesses. Such facilitators typically provide their prospective users with information about the medical interventions businesses are marketing, where clinics are located, and how they can be found online. As a result, research meant to critique the direct-to-consumer marketing of purported stem cell therapies could inadvertently contribute to such commercial activity by being made available on an open access basis. Such data can then be fed to search engines, aggregators, facilitator platforms, and search algorithms that connect businesses engaged in direct-to-consumer marketing of purported stem cell interventions to prospective clients. The various ways in which open-access databases can be used in ways other than their intended academic purposes highlight the urgent need for researchers to discuss what primary source material should be made publicly available, and what information might cause unintended harms if it is made readily accessible.

## Need to Develop Best Practices in Research Methods and Data Archiving

Decisions concerning what research methods to use, whether to archive primary source material, and what archived material to make publicly accessible are connected to the specific questions researchers seek to address. Since researchers often make different choices when identifying research questions worth addressing and determining what forms of evidence need to be gathered, diversity in research methods and attitudes toward archiving of source material is understandable. Acknowledging the merits of taking a pluralistic approach to research methods and methodologies, greater consistency across research teams in the use of social science research methods and data archiving practices might improve scholarly rigor, make it easier to compare and contrast research findings across studies, improve reproducibility of research findings, and help ensure the preservation of primary source materials that might otherwise be lost to public scrutiny.

Collaborative efforts and consensus-based approaches are needed to identify best practices and develop more standardized research approaches and data-archiving practices. However, even before such standards emerge from deliberations involving researchers working in this field of study, there are several compelling reasons why researchers studying direct-to-consumer marketing of purported stem cell treatments should consider making greater use of digital archiving tools to capture and preserve primary source material.

## Electronic Archiving Tools and the Documentation of Direct-to-Consumer Marketing Claims About Purported Stem Cell Therapies

Electronic archiving tools can be used to document and capture digital claims made by businesses engaged in direct-to-consumer marketing of purported stem cell therapies at the time such representations are reviewed and analyzed by researchers [69,70]. Specific examples of such tools include the Wayback Machine, archive.today, archive.is, and archive.md. Additional free and commercially available tools can also be used to archive single web pages or capture digital copies of entire websites. While various tools can be used to archive such digital marketing claims, the Internet Archive’s Wayback Machine is probably the most well-known and widely used online tool for archiving individual web pages and entire websites, and for finding already archived internet content [71,72]. Users can access the Wayback Machine’s archived content and also deposit web pages in it on a cost-free basis, though the Internet Archive accepts donations from users and other supporters.

Since all archiving platforms have vulnerabilities and limitations, I recommend that researchers use more than one method of data archiving, even if they have a preferred tool for creating such repositories. For example, members of my research team and I have used the Wayback Machine as a tool for archiving businesses’ digital marketing representations. However, we complement this approach with print archiving and additional digital archiving of such claims. We focus in particular on capturing marketing representations that businesses make about the purported stem cell products they are selling. Research teams with a different focus might understandably select other representations for digital archiving. For example, researchers studying the sources of evidence businesses use to market purported stem cell interventions might choose to archive marketing content in which businesses refer to published studies or other resources to justify their promotional claims [21].

Numerous electronic archiving tools are available, and researchers studying digital marketing representations made by businesses selling purported stem cell treatments and other regenerative medicine products would benefit from scholarly discussion concerning which tools are most suitable for specific research initiatives. Acknowledging the existence of different archiving tools that can be used to create digital records of web pages and websites, the Wayback Machine internet archive and archiving tool has several features worth noting.

The Wayback Machine has the advantage of allowing users to create digital records of web pages when URLs are uploaded, while also enabling the capture of future versions of company web pages by the platform’s automated web crawlers. Entire websites can also be electronically archived, though websites with a large number of web pages can take time to upload. By uploading URLs at different times, users of the Wayback Machine can help document changes to the marketing claims businesses make over time. Even absent such efforts by users of the Wayback Machine, the Wayback Machine’s automated web crawlers create the potential for future versions of web pages to be captured by the site’s archiving bots. This automated function generates the possibility of digital “snapshots” of company websites being captured and archived after URLs are initially archived. There is no cost to users for uploading web pages to the Wayback Machine and having them electronically archived.

## Case for Increased Digital Archiving of the Online Marketing Claims of Businesses Selling Purported Stem Cell Therapies

Numerous parties could benefit from increased digital archiving of the websites of businesses engaged in direct-to-consumer marketing of purported stem cell treatments.

First, the direct-to-consumer marketplace for companies engaged in direct-to-consumer marketing of purported stem cell therapies is fluid [30,33,36,73]. Many businesses market such purported stem cell treatments for some time and then cease making such advertising claims while continuing to promote other medical interventions. The websites of some once highly visible businesses operating in this marketplace have disappeared from the internet, leaving beyond the Wayback Machine’s archived pages of their company websites few remaining digital traces of their once prominent marketing claims. Creating digital records of web pages when businesses are engaged in direct-to-consumer marketing of purported stem cell treatments provides a way for researchers and journalists to ensure that such promotional claims are captured while they are present on company websites. If researchers do not archive such claims, they risk later finding that such representations have been removed from company websites or placed behind password protected paywalls, or that entire company websites have disappeared from the internet. If pages or entire websites disappear from the internet without first being digitally archived, and if they have not otherwise been captured, it can become impossible to accurately track the history of businesses’ marketing claims.

Digital archiving of online marketing claims can be valuable for researchers who want to be able to provide evidence that they accurately analyzed marketing claims made by businesses that subsequently changed or removed their prior marketing claims from the internet. Such digital archiving can also be crucial if researchers subsequently find themselves faced with legal threats or requests for the retraction or correction of articles when such publications are based on accurate analyses of company websites and there is no legitimate scholarly basis for their removal or revision.

Second, digital archiving of businesses’ websites on a publicly accessible platform such as the Wayback Machine can be helpful to civil litigators, criminal prosecutors, and regulators [74-81] investigating or contesting allegedly deceptive marketing representations or other possible legal violations. Careful analysis of digitally archived web pages can be used to investigate and track, over time, the specific marketing representations businesses use when selling purported stem cell therapies. Documenting continuity and consistency in marketing claims expressly made to clients, or in omitted information that should have been disclosed but was not provided to the customers of such businesses, is important in class action litigation where there must be commonalities that bind together all members of a class of plaintiffs [82]. Digital archiving of businesses’ websites can subsequently enable the introduction of such documentation as evidence in civil litigation or in criminal cases and provide compelling evidence of marketing claims that businesses subsequently attempt to deny or downplay. Such archived material can play an important role in enabling courts to determine what marketing claims businesses made, and whether such representations changed over time.

Just as digitally archived marketing material can play a role in civil litigation, such archived material can also play a valuable role in criminal cases and in the enforcement activities of regulators. While the admissibility in court and legal application of such archived digital representations is likely to be contested by parties who do not want them entered into legal proceedings or who want to challenge how they are used in legal disputes, the existence of such digitally archived material can play a valuable role in documenting the history, substance, and scope of direct-to-consumer marketing claims made by particular businesses.

Third, investigative journalists and other reporters examining the marketing claims, commercial operations, and clinical practices of businesses marketing purported stem cell treatments can benefit from being able to access digitally archived records of company websites. Journalists can use the Wayback Machine to review and analyze the marketing representations made over time by the businesses they are investigating. Being able to access such electronic records is particularly important when such businesses have ceased making such marketing claims, or have altered their marketing representations in significant ways, and reporters must turn to digital archival resources or other sources to reconstruct and document the history of businesses’ marketing claims about purported stem cell treatments. This ability to access archived material is important because businesses can make their websites inaccessible or make changes to website content once they realize they are being investigated by journalists or other parties.

Fourth, digital archives of web pages and websites of businesses engaged in direct-to-consumer marketing of purported stem cell therapies could have important future applications for historians and other researchers studying temporal shifts in the marketing of purported medical therapies [83,84]. The Wayback Machine has already served as a crucial archival resource when conducting a temporal analysis of when 351 US businesses identified in a 2016 study as marketing purported stem cell treatments first began making such digital advertising claims [32,73]. If the direct-to-consumer marketing of purported stem cell treatments is, in time, supplanted by subsequent marketing trends featuring different purported medical therapies such as exosomes, peptides, or “therapeutic” plasma exchange, digital archives of the websites of businesses that engaged in the sale of such putative stem cell treatments might survive as the remaining online traces of such commercial activity. While purported stem cell treatments have now been marketed for approximately twenty years, and there is little reason to think that such promotional claims are about to fade into the past, claims about medical therapies change over time. It is conceivable that what is promoted as “regenerative therapy” years or decades from now could differ in important respects from contemporary marketing representations. For future generations of medical historians, “visiting the archives” will increasingly involve exploring electronic repositories of what once were digital marketing representations made to prospective patients [83,85,86]. Contemporary researchers can play an important role in ensuring that such records are available for future generations of scholars and other interested parties.

Noting the importance of such digital archives, researchers should consider capturing in publicly accessible and searchable archives not only the online direct-to-consumer marketing representations made by businesses selling purported stem cell treatments, but also related legal filings and exhibits from actions taken by the Federal Trade Commission, the Department of Justice, state attorneys general offices, state medical boards, and plaintiffs from successful civil litigation. Such documents could be collected, organized, and made publicly searchable, just as large archives of tobacco industry and opioid industry documents can now be accessed as a result of settlement agreements reached following litigation against businesses selling such products [87-89]. Public access to such troves of primary source material could play a valuable role in the identification of marketing techniques and strategies used by many different businesses operating in this space. It could also provide insight into the legal tactics that such businesses use in response to actions taken by law enforcement bodies and other entities. Textbox 1 summarizes various arguments for digitally archiving marketing claims made by businesses selling purported stem cell therapies on a direct-to-consumer basis.

Textbox 1. **Reasons for digitally archiving the online marketing claims of businesses engaged in direct-to-consumer marketing of purported stem cell treatments.**

**Marketplace fluidity**

- Many businesses over time change the digital marketing claims they make, remove pages, add content to their websites, or remove entire websites from the internet. Digitally archiving individual pages and entire websites helps ensure there is a record of marketing representations businesses have made over time.

**Preserving primary source material**

- Digital archiving of online marketing representations enables researchers to provide evidence that they have accurately analyzed marketing claims made by businesses selling purported stem cell treatments. Digitally archiving such material can help protect researchers from unwarranted demands for corrections or retractions, and from legal threats meant to intimidate researchers critically examining businesses selling unproven and unapproved stem cell interventions.

**Legal actions**

- Digital archiving of businesses’ marketing claims can be helpful to plaintiffs’ attorneys, criminal prosecutors, regulators, and other parties investigating and contesting reportedly deceptive marketing representations and other possible legal violations. Digital archiving of marketing content can be used to document the claims businesses used when advertising stem cell products. Such material can play an important role in civil litigation, criminal prosecutions, and other legal actions.

**Investigative journalism**

- Digital archives containing the history of businesses’ marketing representations can be useful to investigative journalists and other reporters examining the marketing claims specific businesses have made over time. Such digitally archived material can be particularly useful if businesses change their marketing claims or make their websites inaccessible after becoming aware that they are being investigated by journalists.

**Historical research**

- Digital archives of the web pages of businesses that have engaged in selling stem cell products could have valuable future applications for historians and other researchers studying temporal shifts in the marketing of medical interventions.

**Marketing claims and legal strategies**

- Digital archives of marketing claims and related legal filings from legal and regulatory actions taken by the Federal Trade Commission, state attorneys general offices, and other bodies can play an important role in providing public access to the marketing techniques and legal strategies used by businesses selling unapproved and unproven stem cell interventions.

## Limitations of Using the Wayback Machine as a Digital Archiving Tool

While acknowledging the Wayback Machine’s value as an electronic archive, the platform has some limitations that researchers will need to consider when considering what digital archiving tools to use. The Internet Archive, which hosts the Wayback Machine, was hacked and incurred a data breach in which the names, email addresses, and passwords of more than 31 million users of the site were compromised. It is possible the site will be hacked again, and additional data breaches will occur. Other digital archives are also vulnerable to being hacked and to exposing the identities of users.

Because of the web content it archives, the site is sometimes confronted with takedown notifications and lawsuits related to claims of copyright infringement [90]. Other internet archiving sites based outside the United States might be more resistant to such takedown requests and litigation. While the Wayback Machine has, to date, survived such legal disputes, future litigation could conceivably lead to the shuttering of the entire website. Parties that do not want to have their online content archived by the Wayback Machine can submit takedown notices and have archived web pages removed from the archive. Information that has already been archived on the Wayback Machine can be removed following such takedown notices. Furthermore, some websites have automated “bot blockers” that prevent the Wayback Machine’s web crawlers from digitally archiving web pages and websites. As a result, not all web pages submitted to the Wayback Machine for electronic archiving can be digitally archived. These limitations make the Wayback Machine an incomplete and imperfect internet archive, though it still has considerable value as a digital archiving tool and repository.

Acknowledging the Wayback Machine’s limitations, I recommend that researchers analyzing direct-to-consumer marketing representations of purported stem cell treatments use complementary tools when creating archival records of the businesses they have identified and studied. For example, researchers can create digitally archived copies of web pages and websites that are not deposited in a publicly accessible site. Numerous tools can be used to archive such information. Such digital records are not as vulnerable as the Wayback Machine because they are not in the public domain and not, for example, subject to takedown notices.

## Conclusions

Acknowledging the site’s limitations and vulnerabilities, the Wayback Machine has many useful applications when used as a tool for creating an archival record of the digital marketing claims of businesses engaged in direct-to-consumer marketing of purported stem cell treatments. As a researcher working in this area, I have found the Wayback Machine to be a helpful resource when studying the history of digital marketing claims made by businesses engaged in direct-to-consumer advertising of purported stem cell treatments. The Wayback Machine is a particularly useful archival tool to use when investigating how businesses that have disappeared from the internet—whether as a result of civil litigation, regulatory action, criminal charges, marketplace disruptions such as COVID-19, or other factors—once marketed purported stem cell therapies. For example, the archived websites of some of the earliest international businesses to market purported stem cell treatments on a direct-to-consumer basis can still be examined on the Wayback Machine. Visiting the archived websites of these businesses provides insight into what shape marketing representations took when such companies began operating in the early 2000 s [1-3]. It also enables historical research into changes and continuities in digital marketing representations, business operations, and clinical activities.

Members of my research team and I have contributed to the Wayback Machine’s digital archiving of key web pages of businesses engaging in direct-to-consumer marketing of putative stem cell therapies. While I no longer list the names and websites of such businesses in peer-reviewed publications analyzing direct-to-consumer marketing of stem cell interventions due to the risk of legal threats and because of significant concerns about how such lists of businesses can be misused by companies trying to facilitate access to unproven and unapproved stem cell interventions, my team and I have participated in the process of digitally archiving key marketing representations made by businesses engaged in the direct-to-consumer marketing of purported stem cell treatments. I encourage other researchers studying such businesses to consider using the Wayback Machine for this purpose, and to take advantage of other tools for archiving such digital marketing representations. Such efforts should occur as part of a broader effort by scholars working in this area to develop best practices in conducting research on digital advertising claims, archiving primary source material, and establishing ethical frameworks for providing access to archived resources.

## Abbreviations

ISSCR: The International Society for Stem Cell Research

## References

[1] Baker M (2005). Stem cell therapy or snake oil?. Nat Biotechnol.

[2] Enserink M (2006). Selling the stem cell dream. Science.

[3] Kiatpongsan S, Sipp D (2009). Monitoring and regulating offshore stem cell clinics. Science.

[4] Lau D, Ogbogu U, Taylor B, Stafinski T, Menon D, Caulfield T (2008). Stem cell clinics online: the direct-to-consumer portrayal of stem cell medicine. Cell Stem Cell.

[5] Regenberg AC, Hutchinson LA, Schanker B, Mathews DJH (2009). Medicine on the fringe: stem cell-based interventions in advance of evidence. Stem Cells.

[6] Kingery MT, Schoof L, Strauss EJ, Bosco JA, Halbrecht J (2020). Online direct-to-consumer advertising of stem cell therapy for musculoskeletal injury and disease: misinformation and violation of ethical and legal advertising parameters. J Bone Joint Surg Am.

[7] Erikainen S, Couturier A, Chan S (2020). Marketing experimental stem cell therapies in the UK: biomedical lifestyle products and the promise of regenerative medicine in the digital era. Sci Cult (Lond).

[8] Petersen A, Seear K (2011). Technologies of hope: techniques of the online advertising of stem cell treatments. New Genet Soc.

[9] Petersen A, Tanner C, Munsie M (2019). Navigating the cartographies of trust: how patients and carers establish the credibility of online treatment claims. Sociol Health Illn.

[10] Ogbogu U, Rachul C, Caulfield T (2013). Reassessing direct-to-consumer portrayals of unproven stem cell therapies: is it getting better?. Regen Med.

[11] Connolly R, O’Brien T, Flaherty G (2014). Stem cell tourism--a web-based analysis of clinical services available to international travellers. Travel Med Infect Dis.

[12] Nirwan RS, Albini TA, Sridhar J, Flynn HW, Kuriyan AE (2019). Assessing “cell therapy” clinics offering treatments of ocular conditions using direct-to-consumer marketing websites in the United States. Ophthalmology.

[13] Knoepfler PS (2017). The stem cell hard sell: report from a clinic’s patient recruitment seminar. Stem Cells Transl Med.

[14] Hassoun M, Golub S, Greenfield S, Grill JD, Cummings BJ (2022). Online seminars as an information source for direct-to-consumer stem cell therapy. Regen Med.

[15] Taylor PL, Barker RA, Blume KG (2010). Patients beware: commercialized stem cell treatments on the web. Cell Stem Cell.

[16] Ramkumar PN, Navarro SM, Haeberle HS (2017). Cellular therapy injections in today’s orthopedic market: a social media analysis. Cytotherapy.

[17] Kamenova K, Reshef A, Caulfield T (2014). Representations of stem cell clinics on Twitter. Stem Cell Rev Rep.

[18] Chavez J, Shah NA, Ruoss S, Cuomo RE, Ward SR, Mackey TK (2021). Online marketing practices of regenerative medicine clinics in US-Mexico border region: a web surveillance study. Stem Cell Res Ther.

[19] Hawke B, Przybylo AR, Paciulli D, Caulfield T, Zarzeczny A, Master Z (2019). How to peddle hope: an analysis of YouTube patient testimonials of unproven stem cell treatments. Stem Cell Reports.

[20] Kalavar M, Lovett EA, Nicholas MP (2024). Update on “cell therapy” clinics offering treatments of ocular conditions using direct-to-consumer marketing websites in the U.S. Am J Ophthalmol.

[21] Cook M, Richey A, Brafman DA, Frow EK (2021). Weighing up the evidence used by direct-to-consumer stem cell businesses. Stem Cell Reports.

[22] Berger I, Ahmad A, Bansal A, Kapoor T, Sipp D, Rasko JEJ (2016). Global distribution of businesses marketing stem cell-based interventions. Cell Stem Cell.

[23] Munsie M, Pera M (2014). Regulatory loophole enables unproven autologous cell therapies to thrive in Australia. Stem Cells Dev.

[24] Lysaght T, Lipworth W, Hendl T (2017). The deadly business of an unregulated global stem cell industry. J Med Ethics.

[25] McLean AK, Stewart C, Kerridge I (2015). Untested, unproven, and unethical: the promotion and provision of autologous stem cell therapies in Australia. Stem Cell Res Ther.

[26] Munsie M, Lysaght T, Hendl T, Tan HYL, Kerridge I, Stewart C (2017). Open for business: a comparative study of websites selling autologous stem cells in Australia and Japan. Regen Med.

[27] Lysaght T, Munsie M, Hendl T, Tan L, Kerridge I, Stewart C (2018). Selling stem cells with tokens of legitimacy: an analysis of websites in Japan and Australia. Cytotherapy.

[28] Fujita M, Hatta T, Ozeki R, Akabayashi A (2016). The current status of clinics providing private practice cell therapy in Japan. Regen Med.

[29] Kashihara H, Nakayama T, Hatta T, Takahashi N, Fujita M (2016). Evaluating the quality of website information of private-practice clinics offering cell therapies in Japan. Interact J Med Res.

[30] Ikonomou L, Munsie M, Power C, Sipp D, Turner L, Rasko JEJ (2024). Effective regulatory responses to predatory stem cell markets in Australia and Canada. Cell Stem Cell.

[31] Ogbogu U, Du J, Koukio Y (2018). The involvement of Canadian physicians in promoting and providing unproven and unapproved stem cell interventions. BMC Med Ethics.

[32] Turner L, Knoepfler P (2016). Selling stem cells in the USA: assessing the direct-to-consumer industry. Cell Stem Cell.

[33] Turner L (2021). The American stem cell sell in 2021: U.S. businesses selling unlicensed and unproven stem cell interventions. Cell Stem Cell.

[34] Solis MA, Quintana M, Candanedo P JE (2026). Unproven stem cell-based interventions marketed as treatments in Panama: an urgent call for enforcement actions. Stem Cell Reports.

[35] Dulak J, Pecyna M (2023). Unproven cell interventions in Poland and the exploitation of European Union law on advanced therapy medicinal products. Stem Cell Reports.

[36] Knoepfler PS (2019). Rapid change of a cohort of 570 unproven stem cell clinics in the USA over 3 years. Regen Med.

[37] Julian K, Yuhasz N, Hollingsworth E, Imitola J (2018). The “growing” reality of the neurological complications of global “stem cell tourism”. Semin Neurol.

[38] Julian K, Yuhasz N, Rai W, Salerno JA, Imitola J (2020). Complications from “stem cell tourism” in neurology. Ann Neurol.

[39] Bauer G, Elsallab M, Abou-El-Enein M (2018). Concise review: a comprehensive analysis of reported adverse events in patients receiving unproven stem cell-based interventions. Stem Cells Transl Med.

[40] Boudreault K, Justus S, Lee W, Mahajan VB, Tsang SH (2016). Complication of autologous stem cell transplantation in retinitis pigmentosa. JAMA Ophthalmol.

[41] Berkowitz AL, Miller MB, Mir SA (2016). Glioproliferative lesion of the spinal cord as a complication of “stem-cell tourism”. N Engl J Med.

[42] Kuriyan AE, Albini TA, Townsend JH (2017). Vision loss after intravitreal injection of autologous “stem cells” for AMD. N Engl J Med.

[43] Saraf SS, Cunningham MA, Kuriyan AE (2017). Bilateral retinal detachments after intravitreal injection of adipose-derived 'stem cells' in a patient with exudative macular degeneration. Ophthalmic Surg Lasers Imaging Retina.

[44] Wolf AB, Money KM, Chandnani A (2023). Mycobacterium abscessus meningitis associated with stem cell treatment during medical tourism. Emerg Infect Dis.

[45] Brinsfield TN, Pinson NR, Levine AD (2024). The evolution and ongoing challenge of unproven cell-based interventions. Stem Cells Transl Med.

[46] Horner C, Tenenbaum E, Sipp D, Master Z (2018). Can civil lawsuits stem the tide of direct-to-consumer marketing of unproven stem cell interventions. NPJ Regen Med.

[47] Murdoch B, Zarzeczny A, Caulfield T (2018). Exploiting science? A systematic analysis of complementary and alternative medicine clinic websites’ marketing of stem cell therapies. BMJ Open.

[48] Lindvall O, Hyun I (2009). Medical innovation versus stem cell tourism. Science.

[49] Sipp D (2012). Pay-to-participate funding schemes in human cell and tissue clinical studies. Regen Med.

[50] Olsen RJ, Modi IT, Ramkumar PN (2025). Editorial commentary: digital internet information on orthobiologic treatments is low quality, misleading, and unreliable. Arthroscopy.

[51] Kawam O, Zhu X, Watson S (2025). Factors that influence unproven stem cell intervention seeking behavior: a qualitative analysis of U.S. patients considering or having undertaken unproven stem cell interventions. Soc Sci Med.

[52] Kawam O, Zhu X, Eton DT (2023). Designing persuasive health education for patients seeking unproven stem cell interventions. Stem Cell Reports.

[53] Marcon AR, Murdoch B, Caulfield T (2017). Fake news portrayals of stem cells and stem cell research. Regen Med.

[54] Arthurs JR, Martin Lillie CM, Master Z, Shapiro SA (2022). The direct to consumer stem cell market and the role of primary care providers in correcting misinformation. J Prim Care Community Health.

[55] Sipp D (2019). Stem cell mismarketing: implications for the transfusion community. ISBT Sci Ser.

[56] Lovell-Badge R, Anthony E, Barker RA (2021). ISSCR guidelines for stem cell research and clinical translation: the 2021 update. Stem Cell Reports.

[57] Hyun I, Lindvall O, Ahrlund-Richter L (2008). New ISSCR guidelines underscore major principles for responsible translational stem cell research. Cell Stem Cell.

[58] Daley GQ, Hyun I, Apperley JF (2016). Setting global standards for stem cell research and clinical translation: the 2016 ISSCR guidelines. Stem Cell Reports.

[59] Caulfield T, Sipp D, Murry CE, Daley GQ, Kimmelman J (2016). Confronting stem cell hype. Science.

[60] Hinkle JW, Mahmoudzadeh R, Kuriyan AE (2021). Cell-based therapies for retinal diseases: a review of clinical trials and direct to consumer “cell therapy” clinics. Stem Cell Res Ther.

[61] Smith M, Goodheart K, Fryer C, Balcytis T, Wilmshurst P, Murray P (2024). An analysis of the UK’s commercial stem cell clinics. Prometheus.

[62] Kamel S, Smith KR (2021). Stem-cell clinics in the UK: a web-based study. Regen Med.

[63] Turner L (2018). Direct-to-consumer marketing of stem cell interventions by Canadian businesses. Regen Med.

[64] Mole B (2017). The curious case of ClinicalTrials.gov, where dubious stem cell therapies seem legit. Ars Technica.

[65] Centeno C (2023). The sordid tale of the “world’s most advanced” Regen Med procedures. Regenexx.

[66] Knoepfler P (2017). Stem cell clinic-related biz threatens researcher with lawsuit, wants retraction. The Niche.

[67] Ledford H (2011). Stem-cell scientists grapple with clinics. Nature.

[68] Weissman I (2009). The ISSCR: who are we and where are we going?. Cell Stem Cell.

[69] Lepore J (2015). The Cobweb: Can the internet be archived?. The New Yorker.

[70] Dougherty M, Meyer ET (2014). Community, tools, and practices in web archiving: the state-of-the-art in relation to social science and humanities research needs. J Assoc Inf Sci Technol.

[71] Ogden J, Summers E, Walker S (2024). Know(ing) infrastructure: the Wayback Machine as object and instrument of digital research. Convergence.

[72] Arora SK, Li Y, Youtie J, Shapira P (2016). Using the Wayback Machine to mine websites in the social sciences: a methodological resource. J Assoc Inf Sci Technol.

[73] Knoepfler PS, Turner LG (2018). The FDA and the US direct-to-consumer marketplace for stem cell interventions: a temporal analysis. Regen Med.

[74] Eltgroth DR (2009). Best evidence and the Wayback Machine: toward a workable authentication standard for archived internet evidence. Fordham Law Rev.

[75] Evetts M, Bevan H (2022). Wayback evidence: admissible or inadmissible?. Intellect Prop Forum.

[76] Fagan M (2007). “Can you do a Wayback on that?” The legal community’s use of cached web pages in and out of trial. BU J Sci Technol Law.

[77] Soars J, Lee C (2015). Tweets, texts, forged emails and changing webpages: emerging issues and practical examples. Law Soc J.

[78] Gazaryan K (2013). Authenticity of archived websites: the need to lower the evidentiary hurdle is imminent. Rutgers Comput Technol Law J.

[79] Cole J (2016). The brave new world of internet evidence: it’s not as brave or new as it seems. Litigation.

[80] Baker TB (2010). Informal discovery on the internet. Advocate.

[81] Quarles JL, Crudo R (2014). [Way]back to the future: using the Wayback Machine in patent litigation. Landslide.

[82] Seiner JA (2016). Commonality and the constitution: a framework for federal and state court class actions. Indiana Law J.

[83] Owens T, Padilla T (2021). Digital sources and digital archives: historical evidence in the digital age. Int J Digit Humanities.

[84] Knoblauch H, Tomes N (2014). The history of medicine in the digital age. Bull Hist Med.

[85] Carbajal IA, Caswell M (2021). Critical digital archives: a review from archival studies. Am Hist Rev.

[86] Jensen HS (2021). Digital archival literacy for (all) historians. Media Hist.

[87] Bero L (2003). Implications of the tobacco industry documents for public health and policy. Annu Rev Public Health.

[88] Malone RE, Balbach ED (2000). Tobacco industry documents: treasure trove or quagmire?. Tob Control.

[89] MacKenzie R, Hawkins B, Klein DE, Ahmad M, Norman AK, Koon AD (2023). The opioid industry document archive: new directions in research on corporate political strategy. Int J Drug Policy.

[90] Patel K (2007). Authors v. archivers: the copyright infringement battle over web pages. J Pat Trademark Off Soc.

